# Stem cell-derived tissue-associated regulatory T cells ameliorate the development of autoimmunity

**DOI:** 10.1038/srep20588

**Published:** 2016-02-05

**Authors:** Mohammad Haque, Jianyong Song, Kristin Fino, Praneet Sandhu, Xinmeng Song, Fengyang Lei, Songguo Zheng, Bing Ni, Deyu Fang, Jianxun Song

**Affiliations:** 1 Department of Microbiology and Immunology, The Pennsylvania State University College of Medicine, Hershey, PA 17033, USA; 2 Institutes of Irradiation/Immunology, The Third Military Medical University, Chongqing 400038, China; 3 Department of Medicine, The Pennsylvania State University College of Medicine, Hershey, PA 17033, USA; 4 Department of Pathology, Northwestern University Feinberg School of Medicine, Chicago, IL 60611, USA

## Abstract

Pluripotent stem cells (PSCs) have the potential to produce almost all of the cells in the body, including regulatory T cells (T_regs_). However, the exact conditions required for the development of antigen (Ag)-specific T_regs_ from PSCs (*i.e.*, PSC-T_regs_) are not well delineated. Ag-specific PSC-T_regs_ can be tissue/organ-associated and migrate to local inflamed tissues/organs to suppress the autoimmune response after adoptive transfer, thereby avoiding potential overall immunosuppression from non-specific T_regs_. In this study, we developed a new approach to generate functional Ag-specific T_regs_ from induced PSCs (iPSCs), *i.e.*, iPSC-T_regs_, which had the ability to generate an Ag-specific immunosuppressive response in a murine model of arthritis. We retrovirally transduced murine iPSCs with a construct containing genes of Ag-specific T cell receptor (TCR) and the transcriptional factor FoxP3. We differentiated the iPSCs into Ag-specific iPSC-T_regs_ using *in vitro* or *in vivo* Notch signaling, and demonstrated that adoptive transfer of such T_regs_ dramatically suppressed autoimmunity in a well-established Ag-induced arthritis model, including the inflammation, joint destruction, cartilage prostaglandin depletion, osteoclast activity, and Th17 production. Our results indicate that PSCs can be used to develop Ag-specific T_regs_, which have a therapeutic potential for T_reg_-based therapies of autoimmune disorders.

Regulatory T cells (T_regs_) are a component of the normal immune system and contribute to the maintenance of peripheral tolerance. T_regs_ are a subset of specialized CD4^+^ helper T (Th) cells defined phenotypically by the expression of the IL-2 receptor α-chain (CD25) and the transcription factor FoxP3, which is required for T_reg_ development and controls a genetic program specifying this cell fate. T_regs_ can down-regulate immune responses and are essential for immune homeostasis^1^. T_regs_ are key effectors in preventing and treating autoimmune disorders, *e.g.*, rheumatoid arthritis (RA), type-1 diabetes (T1D), and inflammatory bowel diseases (IBD), and controlling both transplant rejection and graft-versus-host disease (GVHD)^2^,^3^,^4^,^5^.

T_reg_-based immunotherapy is a highly promising treatment for a variety of autoimmune disorders, and facilitation for organ transplantation^2^. Although a precise understanding of the immunosuppressive mechanism of T_regs_ remains indefinable, there is increasing evidence that T_regs_ manifest their function through a myriad of mechanisms that include the secretion of immunosuppressive soluble factors (*e.g.*, TGF-β, IL-10, and IL-35), cell contact mediated regulation *via* the high affinity TCR and other membrane-bound molecules (*e.g.*, CTLA-4, GITR, LAG-3), and cytolytic activity. Recent advances in the use of large-scale *in vitro* expansion of T_regs_ followed by *in vivo* re-infusion of these cells raise the possibility that this strategy may be successfully utilized for the treatment of autoimmune disorders^6^,^7^. Although polyclonally expanded populations of T_regs_ exhibit suppressive activity, Ag-specific T_regs_ appear superior in suppressing local autoimmune disorders such as RA, autoimmune diabetes and GVHD^8^,^9^,^10^,^11^,^12^. In addition, tissue/organ (*e.g.*, joints, pancreas, intestine)-associated targeting of T_regs_ stables FoxP3 expression and avoids induction of a potentially detrimental systemic immunosuppression^13^,^14^. For T_reg_-based immunotherapy, the *in vitro* generation of tissue/organ-associated and non-terminally differentiated effector T_regs_ for *in vivo* re-infusion is an optimal approach. However, current methodologies are limited in terms of the capacity to generate, isolate, and expand a sufficient quantity of such T_regs_ from patients for therapeutic interventions.

Under the right circumstance, PSCs can produce almost all of the cells in the body, including T_regs_. PSCs provide a chance to obtain a renewable source of healthy T_regs_ to treat a wide array of autoimmune disorders. However, the right circumstances for the development of antigen (Ag)-specific T_regs_ from PSCs (*i.e.*, PSC-T_regs_) has not been fully defined, especially the signaling mechanisms that direct differentiation of such PSC-T_regs_. Previous studies have shown the successful T cell development from PSCs (5,6), and we have shown T lineage differentiation from iPSCs (7). However, it remains unclear whether iPSCs can differentiate into functional Ag-specific T_regs_ for T_reg_-based immunotherapy.

In this study, we developed a new approach to generate Ag-specific T_regs_ from iPSCs, *i.e.*, iPSC-T_regs_, which have the ability to suppress autoimmunity in a murine model of arthritis. We retrovirally transduced murine iPSCs with a DsRed reporter construct containing genes of major histocompatibility complex (MHC) II (I-A^b^)-restricted ovalbumin (OVA)-specific TCR and the transcriptional factor FoxP3. We differentiated the DsRed^+^ iPSCs into OVA-specific iPSC-T_regs_ with an OP9 stromal cell line expressing Notch ligands DL1, DL4 and I-A^b^ in the presence of recombinant cytokines of rIL-7 and rFlt3L. We visualized the expression of CD3, TCR, CD4, CD25, and CTLA4 on OVA-specific iPSC-T_regs_. Adoptive transfer of such T_regs_ dramatically suppressed autoimmunity in a well-established Ag-induced arthritis (AIA) model, including the inflammation, joint destruction, cartilage proteoglycan depletion, and osteoclast activity. In addition, we measured the IL-17 cytokine secretion from the lymph nodes draining into the joints. We found that FoxP3 and TCR-transduced T_regs_ reduced the number of Th17 producing cells in the OVA-injected knees, where inflammation, bone destruction, cartilage depletion, and osteoclast-like activity were also reduced. Our results indicate that iPSC-derived Ag-specific T_regs_ have a great potential to be used for the treatment of autoimmune disorders.

## Results

### Generation of Ag-specific iPSC-T_regs_

**Figure 1:**
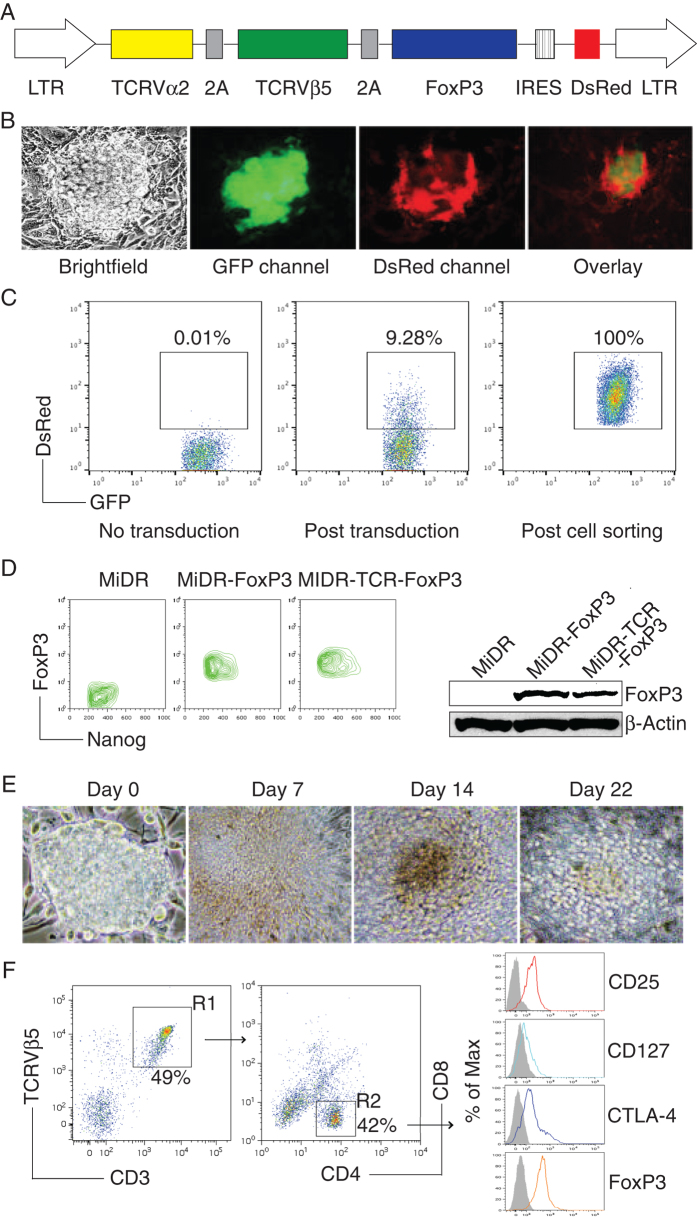
*In vitro* programming of Ag-specific iPSC-T_regs_. (**A**) Schematic representation of the retrovirus construct MiDR-TCR-FoxP3 expressing OVA-specific TCR and FoxP3. Ψ, packaging signal; 2A, picornavirus self-cleaving 2A sequence; LTR, Long terminal repeats. (**B**) The TCR/FoxP3 gene-transduced iPSCs were visualized by fluorescence microscopy. (**C**) GFP^+^ iPSCs (left) were transduced with the retroviral construct MiDR-TCR-FoxP3 and GFP^+^DsRed^+^ iPSCs (middle) were analyzed by Flow Cytometry and sorted by a high speed cell sorter (Right). (**D**) The GFP^+^DsRed^+^ iPSCs were analyzed for the expression of FoxP3 and Nanog by intracellular staining. Data are representative of three independent experiments (left). The GFP^+^DsRed^+^ iPSCs were analyzed using Western blotting (right). Data are representative of three independent experiments. (**E**) Morphology of T_regs_ cell differentiation on day 0, 7, 14 and 22. (**F**) Flow cytometric analysis for the protein expression of iPSC-derived cells on day 28. CD3^+^ TCRVβ5^+^ cells were gated as indicated (R1), and analyzed for the expression of CD4 and CD8, with CD25, CD127, CTLA-4, and FoxP3 shown for cells gated as CD4^+^CD8^-^ cells (R2) (dark lines; shaded areas indicate isotype controls). Data are representative of three experiments.

Previously we have shown that iPSCs have the ability to differentiate into T_regs_^15^, so we sought to investigate whether iPSCs can be induced to differentiate into Ag-specific T_regs_. We used the MiDR retrovirus-mediated transduction and transduced mouse iPSCs with MHC II (I-A^b^)-restricted OVA_323–339_-specific TCR and FoxP3 (MiDR-TCR-FoxP3, Fig. 1A), followed by a co-culture with OP9 stromal cells expressing Notch ligands DL1, DL4 and I-A^b^ (OP9-DL1/DL4/I-A^b^) in the presence of recombinant cytokines of rIL-7 and rFlt3L. Upon gene transduction, we visualized the DsRed expression by fluorescence microscopy (Fig. 1B), and sorted GFP^+^DsRed^+^ cells (Fig. 1C). We confirmed FoxP3 expression in the sorted cells by Flow cytometry and Western blotting (Fig. 1D). To determine whether the TCR and FoxP3 gene-transduced iPSCs were capable of undergoing the differentiation of Ag-specific T_regs_, we first analyzed the iPSC morphological growth in the presence of Notch signaling. After 7 days of culture with the OP9-DL1/DL4/I-A^b^ cells, iPSCs differentiated into mesoderm-like cells, and were associated with non-adherent grape-like clusterson on day 14. On day 22, lymphocyte-like cells spread fully in the plate (Fig. 1E). We further analyzed the cell surface markers of the iPSC-derived cells. On day 28 of *in vitro* co-culture, the iPSC-derived cells substantially expressed CD3 and Ag-specific TCR, two T cell markers. The CD3^+^TCRVβ5^+^ population expressed CD4. Most of the CD3^+^TCRVβ5^+^CD4^+^ cells also expressed CD25, CD127, and CTLA-4, which are typically expressed at elevated levels in naturally occurring T_regs_ (nT_regs_) (23,24,25) and in T cells expressing FoxP3 ectopically (26,27). We also determined that FoxP3 expression in the iPSC-derived cells persisted even after long-term *in vitro* stimulation with the Notch ligand as detected by intracellular staining analyzed by flow cytometry (Fig. 1F). Collectively, our results suggest that iPSCs have the ability to differentiate into Ag-specific CD4^+^CD25^+^FoxP3^+^ T_regs_ by the approach of gene transduction of Ag-specific TCR and FoxP3, followed by stimulation with Notch signaling.

### Functional analyses of Ag-specific iPSC-T_regs_

**Figure 2:**
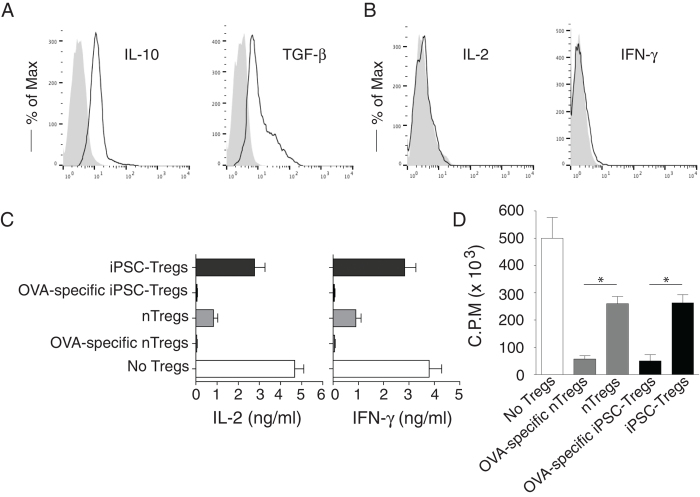
Functional analyses of Ag-specific iPSC-T_regs_. On day 28 of *in vitro* co-culture, the SP CD4^+^TCRβ5^+^ iPSC-T_regs_ were sorted and stimulated by T-depleted splenocytes (APCs; T_regs_/APCs = 1:4) pulsed with OVA_323-339_ peptide (**A,B**), or mixed with naive CD4^+^ T cells (CD4^+^ CD25^-^) from OT-II TCR Tg mice (T_regs_/T cells = 1:10) and then stimulated by the APCs pulsed with OVA_323-339_ peptide for 2 days (**C,D**). The proliferation and cytokine production were assessed. For suppressive assays (**C,D**), a group of CD4^+^ T cells stimulated with OVA-specific nT_regs_ (CD4^+^ CD25^+^) from OT-II TCR Tg mice and a group of CD4^+^ T cells stimulated with non-specific nT_regs_ (CD4^+^ CD25^+^) from C57BL/6 mice as positive controls, and a group of CD4^+^ T cells stimulated without T_regs_ as the negative control. Intracellular cytokine production (IL-10, IL-2, and IFN-γ) or surface LAP (TGF-β) was analyzed by flow cytometry after gating on live CD4^+^ CD25^+^ cells (dark lines; shaded areas indicate isotype controls) or by ELISA, and proliferation was determined by [^3^H] thymidine incorporation. (**A**) Intracellular IL-10 and surface TGF-β. (**B**) Intracellular IL-2 and IFN-γ. (**C**) IL-2 and IFN-γ production were measured by ELISA at 40 h. Data are representative of three experiments. (**D**) Thymidine incorporation during the last 12 hrs. Data are representative of three experiments. *P < 0.05, one-way ANOVA tests.

To determine the functional status of Ag-specific iPSC-T_regs_, we tested whether these iPSC-T_regs_ had the capacity to produce the suppressive cytokines of IL-10 and TGF-β, following Ag stimulation. On day 28 of *in vitro* co-culture, we isolated the CD4^+^CD8^-^ single-positive (SP) iPSC-T_regs_ and stimulated with T-depleted splenocytes pulsed with OVA_323–339_ peptide, and assessed cytokine production. The iPSC-T_regs_ produced LAP (TGF-β) and IL-10 but not IL-2 and IFN-γ as detected by surface or intracellular staining (Fig. 2A,B), indicating that the iPSC-T_regs_ are anergic and have potential suppressive activities.

To further show the functional activity of Ag-specific iPSC-T_regs_, we performed an *in vitro* suppressive assay. We mixed OVA-specific iPSC-T_regs_ on day 28 of the *in vitro* co-culture or nT_regs_ from OT-II TCR Tg mice with naive CD4^+^CD25^−^ T cells (target cells) from C57BL/6 mice (T_regs_/Target cells = 1:10) and stimulated with T-depleted splenocytes pulsed with OVA_323–339_ peptide (T/APCs = 1:4) for 2 days. Supernatants from target cells stimulated with iPSC-T_regs_ or nT_regs_ showed a substantial decrease in the amounts of IL-2 and IFN-γ, as compared to those from target cells alone (Fig. 2C). In a separate set of experiments, effector cells significantly suppressed the proliferation of target cells after OVA peptide stimulation (Fig. 2D). Taken together, these results show that *in vitro*-derived Ag-specific iPSC-T_regs_ are functionally mature.

### *In vivo* programming of Ag-specific iPSC-T_regs_

**Figure 3:**
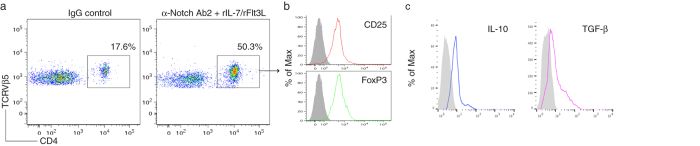
*In vivo* programming of Ag-specific iPSC-T_regs_. The iPSCs transduced with the MiDR-TCR-FoxP3 were co-cultured with the OP9-DL1/DL4/I-A^b^ cells in the presence of murine rFlt3L and rIL-7. On day 7, the DsRed^+^ cells were sorted were adoptively transferred into Thy1.1 congenic mice and in the following days, mice were *i.p.* injected with 0.25 mg agonistic α-Notch2 Ab, 5 μg mouse rIL-7 and 10 μg mouse rFlt3L or a mouse IgG/PBS control twice a week. (**a**) After 2 weeks, CD4^+^ TCRVβ5^+^ cells from the pooled lymph nodes and spleen were analyzed by flow cytometry, after gating on Thy1.2^+^ cell population. (**b**) The expression of CD25 and FoxP3 was analyzed by flow cytometry, after gating on CD4^+^ Thy1.2^+^ TCRVβ5^+^ T cells from the pooled lymph nodes and spleen (dark lines; shaded areas indicate isotype controls). Data are representative of three independent experiments. (**c**) IL-10 and TGF-β production (dark lines; shaded areas indicate isotype controls). The pooled lymph nodes and spleen were stimulated with OVA_323-339_ peptide and analyzed by intracellular IL-10 or surface TGF-β (LAP) staining, after gating on Thy1.2^+^ TCRVβ5^+^ cells. Data are representative of three independent experiments.

Our previous study showed that TCR gene-transduced iPSCs developed into Ag-specific T cells *in vivo*^16^. We tested whether the OVA TCR and FoxP3 gene-transduced iPSCs have the ability to differentiate into OVA-specific T_regs_ *in vivo*. We co-cultured the OVA TCR and FoxP3 gene-transduced iPSCs (Thy 1.2^+^) on the OP9-DL1/DL4/I-A^b^ cells for 7 days, and then adoptively transferred the iPSC-derived cells into the recipient congenic mice (Thy 1.1^+^). We *i.p.* injected agonistic α-Notch2 Ab^17^,^18^ and recombinant cytokines (*e.g.*, rIL-7, rFlt3L) twice a week to boost the development of OVA-specific iPSC-T_regs_ *in vivo*. After 2 weeks, we analyzed CD4^+^ TCRVβ5^+^ cells in the lymph nodes and spleen, gating on Thy1.2^+^ cell population. The approach resulted in approximately 50% of CD4^+^TCRVβ5^+^ cells, gating on Thy1.2^+^ cell population (Fig. 3a), which expressed CD25 and FoxP3 (Fig. 3b), implying that the administration with *in vivo* Notch signaling promotes the development of Ag-specific iPSC-T_regs_. Thy1.2^+^ TCRVβ5^+^ cells from the pooled lymph nodes and spleen were able to respond to Ag stimulation and produced IL-10 and TGF-β (Fig. 3c). These results demonstrate the development of Ag-specific iPSC-T_regs_ using *in vivo* Notch signaling.

### Ag-specific iPSC-T_regs_ ameliorate Ag-induced arthritis (AIA)

**Figure 4:**
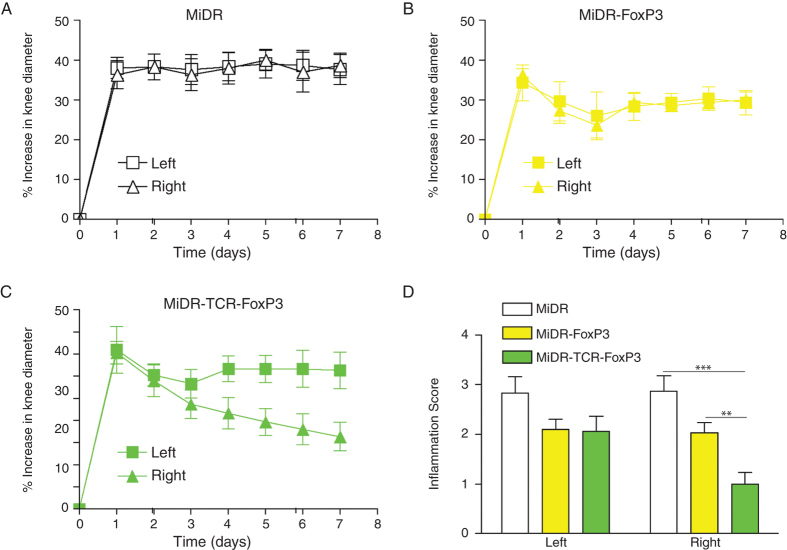
Ag-specific iPSC-T_regs_ ameliorate Ag-induced arthritis (AIA). Murine iPSCs transduced with the retroviral construct MiDR, MiDR-FoxP3, or MiDR-TCR-FoxP3 and were co-cultured on the OP9-DL1/DL4/I-A^b^ cells. On day 7, the gene-transduced cells (3 × 10^6^/mouse) were adoptively transferred into female C57BL/6 mice that were induced AIA two weeks later after the cell transfer. On the following day of arthritis induction, the arthritis severity was monitored by measurement of the knee diameter. (**A–C**) % increase in knee diameter. Increase in knee diameter was calculated based on preinjection knee diameter for each mouse before injection on day 0. Arthritis score was evaluated by examining the both knees by a blinded manner and each knee was assigned a score, as follows: 0, no visible swelling or discoloration; 1, visible swelling with or without discoloration; 2, moderate swelling with discoloration; 3, severe swelling with discoloration. In each group five mice were used and data are representative of three independent experiments. Data are represented as the mean ± SD. (**D**) The mean scoring on day 7 for both knees from five mice. Data are represented as the mean ± SD from three independent experiments (**p < 0.01, ***p < 0.001, two-way ANOVA).

**Figure 5:**
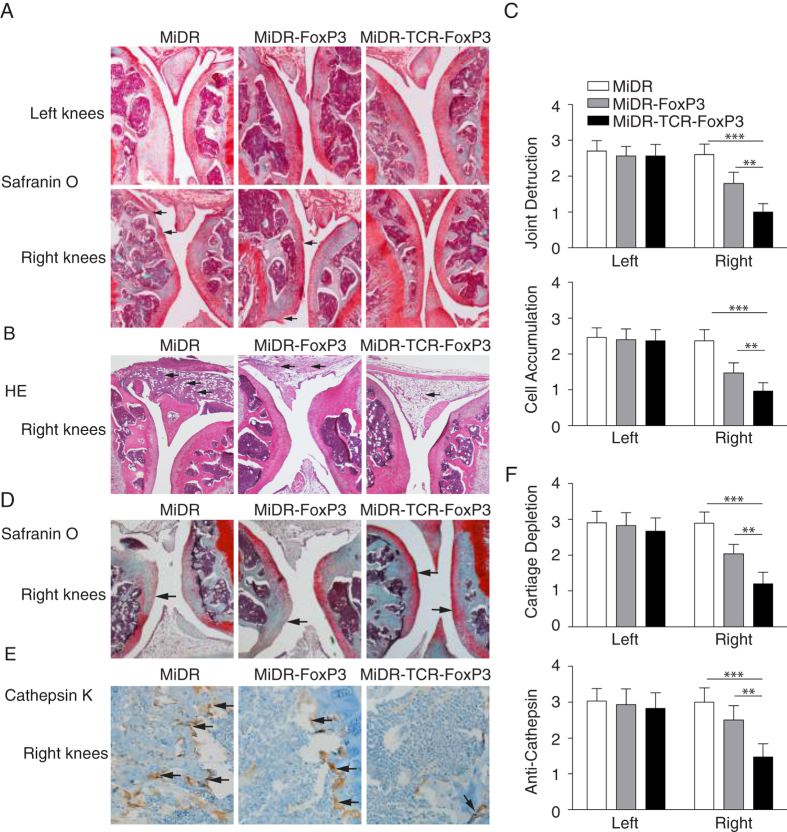
Adoptive transfer of Ag-specific iPSC-T_regs_ ameliorates joint destruction, reduces inflammation and cartilage depletion, and prevents osteoclast-like activity. On day 7 following the arthritis induction, the knees were removed, sectioned and stained with Safranin O, HE, and Cathepsin K for joint destruction, cellular infiltration, cartilage depletion, and osteoclast-like activity. (**A**) Representative photomicrographs of both knees by Safranin O staining. Losses of cartilage and joint destruction (↑) of the right knees are indicated. (**B**) Representative photomicrographs of the right knees by HE staining. Cellular infiltrations (↑) are indicated. Data are representative of five mice per group in three independent experiments. (**C**) Quantitation of joint destruction and cell accumulation from sections of 5 individual knees in each group. Data are represented as the mean ± SD from three independent experiments (*p < 0.05, two-way ANONA). (**D**) Safranin O staining for cartilage depletion. Cartilage depletions (↑) are indicated. (**E**) Cathepsin K staining for osteoclast-like activity. The multinucleated osteoclasts (↑) are indicated. (**F**) Quantitation of cartilage depletion and osteoclast-like activity from sections of 5 individual knees in each group (*p < 0.05, two-way ANOVA).

Therapies that restore or supplement T_reg_ function and number have the ability to inhibit the onset and progression of a number of autoimmune disorders^19^,^20^,^21^,^22^,^23^. To characterize the functional activity of Ag-specific iPSC-T_regs_ in physiological settings, we used the animal protocol wherein murine arthritis was induced in C57BL/6 mice by AIA. In the AIA model, we immunized mice with methylated BSA (mBSA) followed by intra articular knee re-challenge with mBSA to induce T-cell mediated tissue damage^9^. We co-cultured the OT-II TCR and FoxP3 gene-transduced iPSCs on the OP9-DL1/DL4/I-A^b^ cells for 7 days, and then adoptively transferred iPSC-derived cells into the recipient mice that were induced AIA two weeks later after the cell transfer. In the first set of experiments, we tested whether the transferred cells were able to home to the appropriate site, suppress in an Ag-specific fashion, and consequently control joint inflammation locally. We challenged each animal in one knee (right) with mBSA and OVA, and in the contralateral control knee (left) with mBSA alone. As a result, only one knee contained the OVA Ag recognized by the Ag-specific T_regs_, while both knees contained the mBSA Ag recognized by the arthritis-inducing T cells. The results showed that the transferred iPSC-derived cells substantially decreased the inflammatory knee swelling when OVA was present, but had no effect on the control knee that was injected with mBSA only (Fig. 4). In a control group, FoxP3-transduced T cells also reduced inflammation, but had a lower level than the transduced iPSC-derived cells. On day 7, after AIA, we sacrificed mice and removed both knees for histopathological examination to detect *in vivo* tissue damage. Using the Safranin O staining, we observed in the vector control (MiDR) group, there was a loss of cartilage and joint destruction over large areas of the joint surface of both bones. In the MiDR-FoxP3 group^15^, there was a loss of cartilage, but mainly restricted to one bone surface of the joint. In the MiDR-TCR-FoxP3 group, there was no obvious loss of cartilage, showing normal knee architecture with an area of limited cartilage thinning (Fig. 5A,C). Using the HE staining, we identified that compared with the MiDR group in which the right knees showed considerable cellular infiltration over a large proportion of the joint surface, the MiDR-FoxP3 or MiDR-TCR-FoxP3 group had few cellular infiltration areas (Fig. 5B,C). These results showed that lymphocyte infiltration and bone erosion were markedly reduced in the right knees where OVA was present.

In the second set of experiments, we investigated whether Ag-specific iPSC-T_regs_ could suppress cartilage depletion and prevent osteoclast-like activity. In autoimmune arthritis, bone destruction is attributable to excessive bone resorption by osteoclasts, the formation of which is directly and indirectly regulated by CD4^+^ T cells infiltrating into the lesion (29,30). Osteoclasts are multinucleated cells of monocyte/macrophage lineage that degrade bone matrix and dynamically remodel the skeleton (31–33). During autoimmune arthritis, cartilage proteoglycan depletion and osteoclast activity are markedly increases. In the AIA model, we identified the increased cartilage proteoglycan depletion and osteoclast-like activity, which was correlated with the increase of joint inflammation and bone erosion. On day 7, after the AIA, we sacrificed mice and removed both knees for histopathological examination to detect osteoclast-like activity and proteoglycan depletion. Using Safranin O staining, we observed in the vector control (MiDR) group, there was severe cartilage proteoglycan depletion, which was reduced or dramatically reduced in the MiDR-FoxP3 or MiDR-TCR-FoxP3 group, separately (Fig. 5D). Using Cathepsin K staining, we identified that compared with the MiDR group in which the right knees showed a number of cellular infiltration areas with multinucleated osteoclasts, the MiDR-FoxP3 or MiDR-TCR-FoxP3 group had reduced or substantially reduced cellular infiltration areas (Fig. 5E). Our results clearly demonstrated that the iPSC-T_regs_ were successful to reduce cartilage proteoglycan depletion and osteoclast-like activity (Fig. 5F). Of note, in the OVA-injected knee, but not in the PBS treated-knees, reduced the cartilage depletion and osteoclast-like activity existed, suggesting that the iPSC-T_regs_ are Ag-specific and functional, and Ag-specific iPSC-T_regs_ are more efficient in suppressing the AIA than non Ag-specific T_regs_.

Collectively, these results demonstrate that Ag-specific iPSC-T_regs_ are tissue-associated, and are able to migrate into local inflammation areas to prevent the joint damage in the AIA model.

### *In vivo* persistence of Ag-specific iPSC-T_regs_

**Figure 6:**
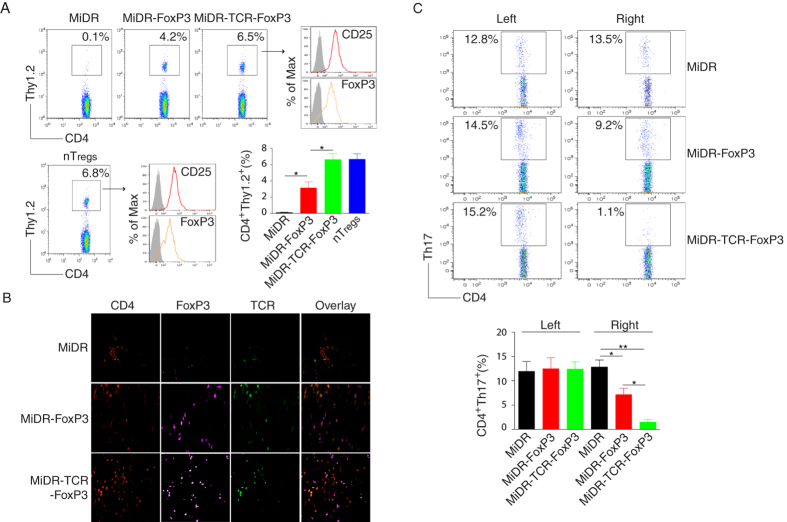
Ag-specific iPSC-T_regs_ infiltrate into the knee joints, maintain the T_reg_ phenotype *in vivo*, and suppress AIA by reducing the local number of Th17 producing cells. (**A**) The iPSC-T_regs_ or nT_regs_ (CD4^+^ CD25^+^) from OT-II TCR Tg mice (Thy 1.2^+^) were adoptively transferred into C57BL/6 congenic mice (Thy1.1^+^) with AIA. Six weeks later, mice were sacrificed and the popliteal lymph nodes from the inflammatory right side were analyzed for CD4^+^ Thy 1.2^+^ cells. Data are representative of three independent experiments. The mean ± SD from three independent experiments is shown (*p < 0.05, one-way ANONA). (**B**) On days 7-14 after arthritis induction, the knees were removed and stained for immunohistology with CD4, FoxP3, and TCRVβ5. Data are representative of five mice per group in three independent experiments. (**C**) On day 14 after arthritis induction, mice were sacrificed and the popliteal lymph nodes were removed from both sides, and the cells were analyzed for intracellular IL-17 staining, gating on CD4^+^ populations. Data are representative of three independent experiments. The mean ± SD from three independent experiments is shown (*p < 0.05, **p < 0.01, one-way ANOVA).

The stability or plasticity of the T_reg_ phenotype and function is a dynamic process modulated by inflammatory signals, T_regs_ may down-regulate FoxP3 and lose regulatory activity^24^. We explored the *in vivo* persistence of Ag-specific iPSC-T_regs_. We co-cultured the OT-II TCR and FoxP3 gene-transduced iPSCs (Thy 1.2^+^) on the OP9-DL1/DL4/I-A^b^ cells for 7 days, and then adoptively transferred the iPSC-derived cells into the recipient congenic mice (Thy 1.1^+^) with AIA. Six weeks later, we sacrificed mice and analyzed the population of CD4^+^ Thy 1.2^+^ cells in the popliteal lymph nodes from the inflammatory right side. Approximately 6.5% of Thy 1.2^+^ cells existed in the recipient mice, gated on live CD4^+^ population, which came from the transferred iPSC-derived cells, similar to the control OVA-specific nT_regs_ from OT-II TCR transgenic (Tg) mice. In addition, these Thy 1.2^+^ cells highly expressed CD25 and FoxP3 (Fig. 6A). This result indicated that the Ag-specific iPSC-T_regs_ have a functional stability.

There is evidence that TCR has a key role in influencing the phenotype, function and localization of T_regs_ *in vivo*^25^,^26^. We analyzed the Ag specificity of OVA-specific iPSC-T_regs_ by locating these cells in the OVA-treated knees. On day 14, after the AIA, we sacrificed mice and removed both knees for histopathological examination. Fluorescence microscopy revealed that more FoxP3^+^ cells were present in the OVA-treated than PBS-treated knees; there was no FoxP3^+^ cells existing in the knees in mice receiving the DsRed^+^ vector-transduced iPSCs. Of note, much more CD4^+^FoxP3^+^ TCRVβ5^+^ cells presented in the knees in mice receiving iPSCs transduced with the MiDR-TCR-FoxP3 than MiDR-FoxP3 (Fig. 6B). These data clearly suggest that the Ag-specific TCR and FoxP3 stably expressing in the iPSC-T_regs_ drove them accumulating in the inflamed joints and efficiently suppressed the AIA.

### Ag-specific iPSC-T_regs_ ameliorate AIA by suppressing the development of IL-17 producing cells

To determine the mechanisms by which Ag-specific iPSC-T_regs_ suppress the development of autoimmune arthritis, we investigated the role of IL-17 producing cells in the AIA model. IL-17 is a pro inflammatory cytokine produced mainly by activated T cells, and plays a pathogenic role in animal arthritis models and in human RA. Neutralizing IL-17 during the induction of collagen-induced arthritis (CIA) showed that IL-17 played a role in the early and late stages of the CIA. We monitored the number of Th17 producing cells in the AIA experiments. We adoptively transferred the iPSC-derived cells into the recipient mice before mBSA re-challenge. There were three groups of iPSC-derived cells: the MiDR vector, MiDR-FoxP3^15^, and MiDR-TCR-FoxP3. Compared to the MiDR and MiDR-FoxP3-treated mice, the MiDR-TCR-FoxP3-treated mice showed a significant reduction of Th17 producing cells (Fig. 6C). There were similar numbers of Th17 producing cells in the PBS control knees (left), indicating that the suppression of Th17 numbers required the presence of the OVA Ag recognized by the iPSC-T_regs_. Of note, the ability to suppress the numbers of Th17 producing cells and swelling correlated with the ability to reduce joint inflammation, bone destruction, and osteoclast-like activity as judged by histopathology of tissue section. Taken together, these results suggested that Ag-specific iPSC-T_regs_ ameliorate the development of AIA by suppressing Th17 producing cells and the suppressive activity is in an Ag dependent fashion.

## Discussion

In this study, we show the generation of functional Ag-specific T_regs_ by reprogramming iPSCs. In addition, we demonstrate that adoptive transfer of Ag-specific iPSC-T_regs_ suppresses the development of AIA. Furthermore, we determine that Ag-specific iPSC-T_regs_ ameliorate the development of AIA by suppressing Th17 producing cells and the suppressive activity is in an Ag dependent fashion. These results show that we develop a novel approach for developing potentially therapeutic Ag-specific T_regs_.

Strong arguments support the development of T_reg_-based therapies in autoimmune disorders using engineered T_regs_. While clinical trials show safety, feasibility, and potential therapeutic activity of T_reg_-based therapies using this approach, concerns about autoimmunity due to cross-reactivity with healthy tissues remains a major safety issue^27^. In addition, genetically modified T_regs_ using current approaches are usually intermediate or later effector T_regs_ that only have short-term persistence *in vivo*^28^,^29^.

There are numerous advantages of T_reg_-based immunotherapy over conventional treatments. These benefits include: (1) the potential for Ag specificity with the lack of general immunosuppression; (2) the possibility of inducing long-lasting regulation *in vivo*, and (3) the custom-made product that can be designed for each patient, with very limited or absent side effects. However, due to the lack of T_reg_ specific surface Ags, purification of a more comprehensive T_reg_ subset increases the contamination by the non-regulatory T_effs_. Of note, in autoimmune prone mice or humans with autoimmune disorders, contamination of therapeutic T_regs_ by non-regulatory T_effs_ can include pathologic autoreactive lymphocytes. In addition, although naive T cells or thymocytes could be easily transduced with retrovirus containing FoxP3^30^,^31^,^32^, the transfer of FoxP3 into the cells does not recapitulate the full repertoire of transcripts and functional characteristics of nT_regs_^33^. Furthermore, the generated T_regs_ are not Ag-specific. Many examples exist that Ag-specific T_regs_ are more effective in autoimmune syndromes when compared to polyclonal populations^34^,^35^,^36^. With this, a major challenge in Ag-specific T_reg_-based immunotherapy lies in the isolation of sufficient quantities of Ag-specific T cells - a challenge that is not easily overcome in humans where sampling is largely limited to the peripheral blood. A new approach by viral TCR gene transfer has been developed to generate Ag-specific T_regs_ from a fully mature polyclonal nT_regs_ population isolated from the peripheral blood mononuclear cells (PBMCs). This approach augmented the therapeutic profile of T_regs_ in an arthritis murine model and redirected T_regs_ *in vivo*^37^. However, TCR transduction of PBMCs will induce a T-cell population with mispaired TCRs, thereby reducing the surface density of Ag-specific TCR^38^. In contrast, transduction of stem cells followed by T cell lineage differentiation by *in vitro* or *in vivo* Notch signaling can overcome this problem^39^,^40^.

To date, PSCs are the only source available to generate a high number of single-type Ag-specific T_regs_^15^,^41^,^42^. The approach to obtain embryonic stem cells (ESCs) from patients is not feasible. Hematopoietic stem cells (HSCs) have a higher potential to pass the bone marrow (BM) barrier and travel in the blood, which allows HSCs to be harvested from the patient blood following mobilization with hematopoietic growth factors. Thus, the use of HSCs for therapeutic purposes has been widely applied in the clinic, especially in HSC transplantations; however, HSCs are similar to other adult stem cells, which can proliferate only for a limited number of cycles. HSCs’ response to differentiation signals decline with each cycle. In other words, compared to ESCs, HSCs have reduced differentiation and proliferative capacities, and the numbers of HSCs are difficult to expand in cell culture^43^. Human iPSCs can be easily generated from patients’ somatic cells by transduction of various transcription factors and exhibit characteristics identical to those of ESCs^44^. Many genetic methods as well as the protein-based approaches have been developed to produce iPSCs with potentially reduced risks, including that of immunogenicity and tumorigenicity^45^. This approach provides an opportunity to generate patient- or disease-specific iPSCs^46^. Because of the plasticity and the potential for an unlimited capacity for self-renewal, iPSCs have high potential for advancing the field of cell-based therapies. Our laboratory has shown that programming of Ag-specific iPSC-CTLs or iPSC-T_regs_ can be used for immunotherapy of cancers and autoimmune disorders^15^,^16^,^42^,^47^; other groups reported related investigations^48^,^49^. In this study, we have developed a system to generate Ag-specific iPSC-T_regs_. Our research provides a new insight into the methodologies and mechanistic requirements for efficient development of stable tissue-associated or organ-specific iPSC-T_regs_. Once such strategies become available, there is the potential to facilitate the generation of immune tolerance.

Although Ag-specific iPSC-T_regs_ have promising therapeutic effects in cell-based therapies, their efficiency is limited by the need to generate a large number of such cells using complex and expensive *in vitro* differentiation. In addition, the lengthy duration for generating iPSCs may limit their use in individualized therapies. Alternatively, the TCR/FoxP3 gene-transduced iPSCs can be used to differentiate Ag-specific iPSC-T_regs_ *in vivo* and suppress autoimmune disorders. After the adoptive transfer of the gene-transduced iPSCs, Notch agonists or recombinant cytokines (*e.g.*, rIL-7, rFlt3L) can be administrated to boost *in vivo* development of Ag-specific iPSC-T_regs_. Another potential problem could arise including risks from the transferred cells potentially differentiating into other cell types (*e.g.*, leukemia, lymphoma) *in vivo* or becoming dysfunctional. Alternatively, we will incorporate a suicide gene, the inducible caspase 9 ^59^, into the vector as this allows the removal of the transduced T_regs_ by the injection of a bioinert small-molecule dimerizing agent (AP1903) to “shut off” the system, which will overcome this potential problem.

For autoimmune disorders like RA, it is unclear which Ags are critical for the disease. Clinical work has been performed targeting the autoAg that are relatively abundantly expressed in the inflamed synovium^50^,^51^,^52^. A clinical trial using heat shock protein (hsp) demonstrated clinical remission in patients with juvenile RA^53^. Recent studies have uncovered potent disease-suppressive T_regs_ recognizing hsp self-Ags, enabling selective activity in inflamed tissues^8^. Generating hsp-specific iPSC-T_regs_ for immunotherapy has a potential in the therapeutic interventions of autoimmune arthritis and lupus.

In summary, we have developed a new approach to generate functional Ag-specific iPSC-T_regs_, which have the ability to suppress autoimmunity in a murine model of arthritis. Targeting tissue/organ-associated iPSC-T_regs_ may drive forward the use of therapeutic T_regs_ of cell-based therapies.

## Methods

### Ethics statement

All animal experiments were approved by the Pennsylvania State University College of Medicine Animal Care Committee (IACUC protocol #2008-015) and were conducted in compliance with the guidelines of the Association for the Assessment and Accreditation of Laboratory Animal Care.

### Cells and mice

The mouse iPS-MEF-Ng-20D-17 cell line (GFP^+^), which was induced from mouse embryonic fibroblasts by retroviral transfection of Oct3/4, Sox2, Klf4, and c-Myc, was obtained from Dr. Shinya Yamanaka (Institute for Frontier Medical Sciences, Kyoto University, Kyoto, Japan) through the RIKEN Cell Bank (Ibaraki, Japan)^16^. The OP9-DL1/DL4/I-A^b^ cell line was generated by a retroviral transduction of the OP9 cells from ATCC (Manassas, VA) with the murine genes of DL1, DL4 and I-A^b^ 15. OT-II TCR-Tg mice, expressing a TCR composed of variable (Vβ5 and Vα2) chains responsive to an OVA peptide 323-339 (ISQAVHAAHAEINEAGR), were bred on a C57BL/6 background. C57BL/6 (Thy1.2) and Thy1.1 congenic mice (B6.PL-*Thy1a*/Cy) mice were purchased from The Jackson Laboratory (Bar Harbor, ME).

### Chemicals, peptide and antibodies

Albumin from chicken egg white (whole OVA, # 5503) and albumin methylated from bovine serum (mBSA, #A1009) were purchased from Sigma-Aldrich (St. Louis, MO). Complete Frenud’s adjuvant (CFA, #263810) was purchased from BD Difco (Sparksm MD). OVA_323-339_ peptide was purchased from the American Peptide Company (Sunnyvale, CA). PE or APC anti-mouse IL-2 (JES6-5H4) IFN-γ (XMG1.2) were obtained from BD Biosciences (San Diego, CA). PE, PE/Cy7, APC, APC/Cy7 or Alexa 647- conjugated anti-mouse CD4 (GK1.5), TCRVβ5 (MR9-4), CD3 (17A2), CD25 (3C7), CTLA-4 (UC10-4B9), FoxP3 (MF-14), IL-10 (JES5-16E3), latency associated peptide/LAP (TGF-β1, TW7-16B4), and Th17A (TC11-18H10.1) were obtained from Biolegend (San Diego, CA). Anti-FoxP3 Ab (ab54501) for immunofluorescent staining was purchased from Abcam (Cambridge, MA). Alexa Fluor 488-conjugated anti-mouse Nanog (eBioMLC-51) were obtained from eBioscoence (San Diego, CA). FITC- or PE-conjugated anti-mouse CD8 (6A242) was obtained from Santa Cruz Biotechnology (Santa Cruz, CA). Rabbit polyclonal anti-Cathepsin K Ab (#11239-1-AP) were purchased from Proteintech group (Chicago, IL). Mouse α-Notch2 Ab was kindly provided by Dr. Hideo Yagita (Juntendo University School of Medicine, Japan)^18^.

### Cell cultures

Mouse iPSCs were maintained on feeder layers of irradiated SNL76/7 cells in 6-well culture plates (Nunc) and passaged every 3 days. In brief, iPSCs were maintained in DMEM culture medium (0.1 mmol/L nonessential amino acids, 1 mmol/L L-glutamine from Invitrogen (Carlsbad, CA), and 0.1 mmol/L β-mercaptoethanol from Sigma-Aldrich (St. Louis, MO) supplemented with 15% fetal calf serum (FCS) from HyClone, (Logan, UT). Monolayers of OP9-DL1/DL4/I-A^b^ cells were cultured in α-MEM medium supplemented with 20% FCS and 2.2 g/L sodium bicarbonate from Invitrogen (Carlsbad, CA). Mouse iPSCs were washed once in DMEM culture medium before plating onto sub confluent OP9-DL1/DL4/I-A^b^ monolayers for T lineage differentiation in the presence of murine rFlt-3 ligand (5 ng/ml) and rIL-7 (1 ng/ml) from Peprotech (Rocky Hill, NJ)^15^.

### Retroviral transduction

cDNA for FoxP3 or Foxp3 with OVA-specific TCR was subcloned into the murine bicistronic retroviral vector MSCV-IRES-DsRed (MiDR)^16^, and cloning was confirmed by PCR amplification and gene sequencing. Retroviral transduction was performed as described^15^.

### Adoptive transfer

DsRed^+^ T_reg_ progenitors (3 × 10^6^) from iPSCs in PBS were injected *i.v.* into 6-wk-old C57BL/6 mice. After 4-6 wk, mice were scarified, and the lymph nodes, spleen, or knees were removed for histopathological examination.

### Flow cytometric analysis

Gene-transduced iPSCs (DsRed^+^) were co-cultured with OP9-DL1/DL4/I-A^b^ cells for various days, and the expression of CD3, TCRβ, CD4, CD25, and CTLA-4 was analyzed by flow cytometry, after gating on DsRed^+^ cells or other markers such as CD3 or TCR. Intracellular IL-10 and surface LAP/TGF-β were analyzed by flow cytometry, after gating on live CD4^+^ CD25^+^ cells or Thy1.2^+^ TCRVβ5^+^ cells. T cells from the popleteal lymph nodes were collected and intracellular Th17 producing cells were analyzed by flow cytometry, after gating on live CD4^+^ cells.

### Cytokine secretion and proliferation

Cytokines (IL-2 and IFN-γ) were measured by ELISA, and proliferation was measured in triplicate cultures by incorporation of ^3^H-thymidine (1 μCi/well; ICN Pharmaceuticals) during the last 12 hours of culture^15^.

### Immunoblot

Live cells were lysed in ice-cold RIPA lysis buffer for 30 min. Insoluble material was removed and lysates were used for Western blotting as described^15^.

### Induction of Ag induced arthritis (AIA)

The gene-transduced iPSCs (DsRed^+^) were co-cultured with OP9-DL1/DL4/I-A^b^ cells for 7 days. The iPSC-derived cells were adoptively transferred to the female C57BL/6 mice through *i.v.* injection (day 0). At day 10, mice were *s.c.* immunized at the base of the tail with 100 μg mBSA emulsified in complete Freund’s adjuvant. At day 20, arthritis was induced by intra-articular injection of 20 μg mBSA in 10 μL PBS in the left knee and 20 μg mBSA and 100 μg whole OVA in 10 μLPBS in the right knee. Arthritis severity was monitored by measurement of knee diameter using a dial gauge caliper from Kroeplin (Schluchtern, Germany). Increase in knee diameter was calculated based on pre-injection knee diameter for each mouse before injection on day 0 as described^9^.

### Histology and immunofluorescence

Mice were sacrificed on day 7 after AIA induction. Both knees were removed, fixed in 10% formalin, and calcified in Formical-4 from Decal Chemical (Tallman, NY). The tissues were embedded in Paraffin, sectioned at 4 μm, and stained with hematoxylin and eosin (HE) or Safranin O staining. Safranin O staining for bone erosion and loss of proteoglycans was scored using a semi quantitative scoring system from 0 to 4. For bone erosion, “0” is considered as no erosion, and “4” is considered as extended erosion and destruction of bone. For proteoglycans depletion, “0” represents no loss of proteoglycans, and “4” indicates a complete loss of staining for proteoglycans. HE staining for infiltration of inflammatory cells was scored using a semi quantitative scoring system as described^15^.

Immunofluorescent staining was performed on the sections after deparaffinization and rehydration as described^15^. Cathepsin K was diluted at 1:100 and incubated for 32 minutes followed by detection and visualization with Ventana Omnimap DAB anti-Rabbit detection kit.

### Statistical analysis

One-way or two-way ANOVA was used for statistical analysis between groups and significance was set at 5%. All statistics were calculated using GraphPad Prism (San Diego, CA).

## Additional Information

**How to cite this article**: Haque, M. *et al.* Stem cell-derived tissue-associated regulatory T cells ameliorate the development of autoimmunity. *Sci. Rep.* **6**, 20588; doi: 10.1038/srep20588 (2016).

## Supplementary Material

Supplementary Information
