# Supplementary material for: Stem cell-derived tissue-associated regulatory T cells ameliorate the development of autoimmunity
Source: Sci Rep. 2016 Feb 5;6:20588. doi: 10.1038/srep20588 (PMC4742827; doi:10.1038/srep20588)
Supplement: Supplementary Information [file srep20588-s1.pdf]

Stem cell-derived tissue-associated regulatory T cells ameliorate the development of autoimmunity

Mohammad Haque[1, #], Jianyong Song[2, #], Kristin Fino[1], Praneet Sandhu[1], Xinmeng Song[1], Fengyang Lei[1], Songguo Zheng[3], Bing Ni[2], Deyu Fang[4], and Jianxun Song[1]

[1] Department of Microbiology and Immunology, The Pennsylvania State University College of Medicine, Hershey, PA 17033, USA
[2] Institutes of Irradiation/Immunology, The Third Military Medical University, Chongqing 400038, China
[3] Department of Medicine, The Pennsylvania State University College of Medicine, Hershey, PA 17033, USA
[4] Department of Pathology, Northwestern University Feinberg School of Medicine, Chicago, IL 60611, USA
[#] These authors contributed equally to this work

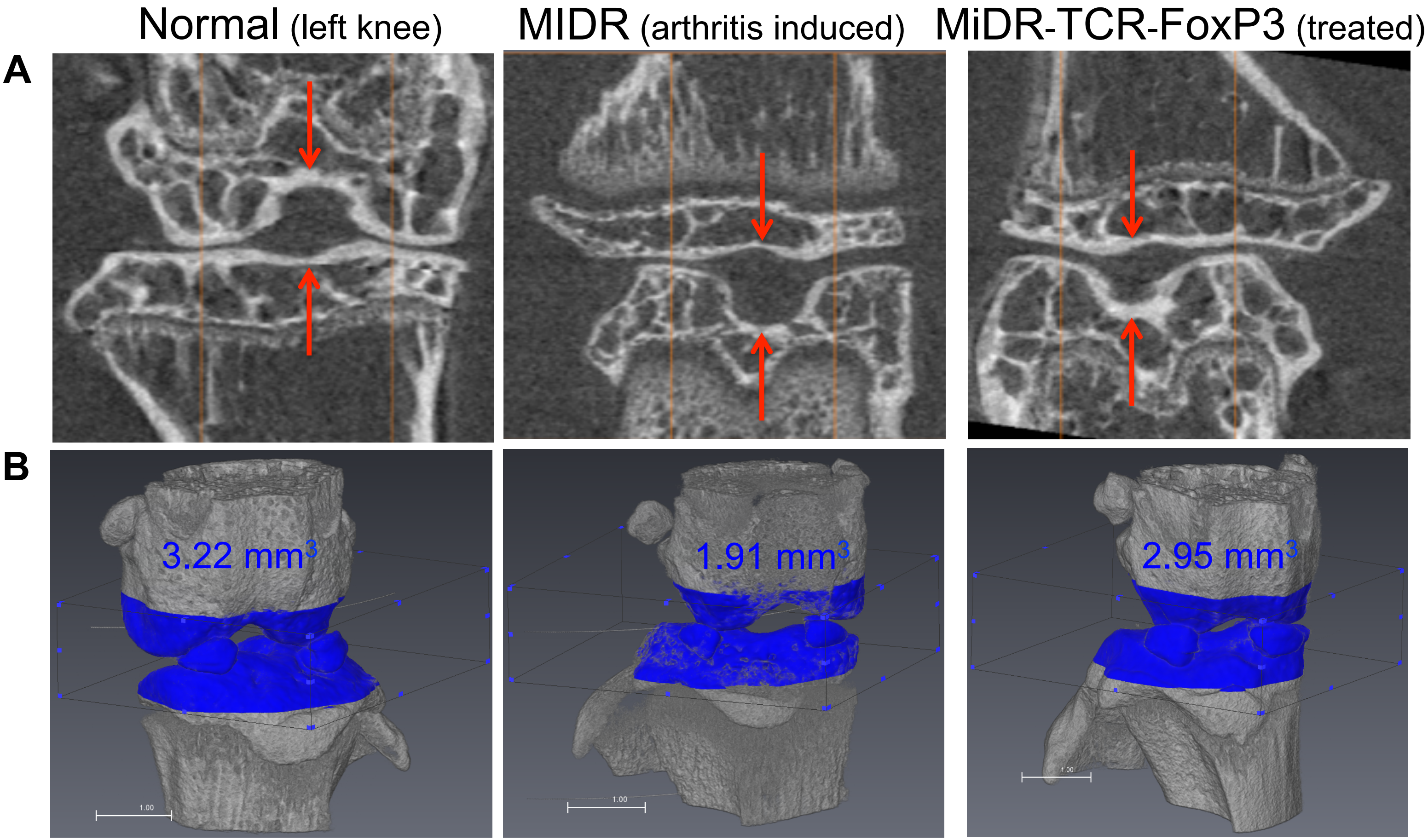

***In vivo* imaging of the bone architecture around the mouse knees by the high-resolution micro-computed tomography (micro-CT) system. (A)** The scans were performed with 2.2 mm length, and images were captured after further image processing (volume rendering and transformation). (**B**) Bone volume around knee joint was evaluated using three dimensional reconstruction of micro-CT images, and bone volume inside the volume of interest was calculated.
